# Evaluation of the epidemiologic triad in the incidence of peritonitis among pediatric patients with chronic kidney disease undergoing peritoneal dialysis: a single-center study in Indonesia

**DOI:** 10.1186/s12887-026-07185-8

**Published:** 2026-06-26

**Authors:** Clarissa Tania, Aaron Tigor Sihombing

**Affiliations:** Department of Urology, Hasan Sadikin Hospital, Universitas Padjadjaran, Jl. Pasteur No.38, Bandung, West Java Indonesia

**Keywords:** Peritonitis, Pediatric, CAPD, Epidemiologic triad, Socioeconomic factors

## Abstract

**Introduction:**

Peritonitis is the most common and serious complication of Continuous Ambulatory Peritoneal Dialysis (CAPD) in children. Understanding the interaction of host, agent, and environmental factors—based on the epidemiologic triad—may help reduce its incidence in pediatric populations, especially in resource-limited settings. This study aimed to evaluate host and environmental factors associated with peritonitis among pediatric CAPD patients while descriptively characterizing the causative microorganisms involved.

**Methods:**

This retrospective study included 35 pediatric patients receiving CAPD from 2019 to 2024. Clinical, socioeconomic, nutritional, and environmental data were collected. Peritonitis diagnosis followed ISPD criteria. Peritonitis incidence and peritonitis-free survival were analyzed using Kaplan–Meier curves and bivariate statistical tests.

**Results:**

Eleven patients (31.4%) developed peritonitis, with an overall rate of 4.5 episodes per 100 person-months. Low dietary diversity, well water consumption, and low paternal education or labor/driver occupations were associated with higher peritonitis occurence (*p* < 0.05). Kaplan–Meier analysis showed that low dietary diversity significantly reduced peritonitis-free survival (*p* = 0.021). Trends were observed for water source and occupation.

**Conclusion:**

Peritonitis in pediatric CAPD patients results from a multifactorial interplay of nutritional, environmental, and socioeconomic factors. Addressing these determinants through dietary support, hygiene education, and socioeconomic assistance may help reduce peritonitis risk and support better outcomes.

**Supplementary Information:**

The online version contains supplementary material available at https://doi.org/10.1186/s12887-026-07185-8.

## Introduction

Children with end-stage renal disease (ESRD) require comprehensive long-term care to support growth, psychosocial development, and quality of life. Kidney transplantation remains the optimal renal replacement therapy for achieving normal growth, psychosocial development, educational attainment, and long-term quality of life in pediatric ESRD patients. However, in resource-limited settings where access to transplantation remains constrained, CAPD serves as an important bridging and maintenance therapy that supports children’s growth, education, and overall development while awaiting definitive treatment [1, 2].

In Indonesia, the past ten years have seen a worrying increase in cases of End-Stage Renal Disease (ESRD) among children. As a result CAPD has become an essential treatment, acting as the main option for pediatric patients waiting for a kidney transplant. This underscores the escalating challenge of childhood ESRD and the importance of CAPD in caring for these young patients until they can receive a transplant that could transform their lives [3]. Chronic kidney disease (CKD) in children can hinder growth and development, thereby compromising long-term goals for child well-being [4].

CAPD has emerged as a more practical alternative with demonstrable benefits to patients’ quality of life [5, 6]. Nevertheless, peritonitis remains the most significant complication of CAPD in the pediatric population. It contributes to considerable morbidity and may result in irreversible technique failure. According to the United States Renal Data System (USRDS), infectious complications are the most frequent cause of hospitalization among children receiving CAPD, with cumulative hospitalization rates over a 36-month period increasing from 19.3% (1995–1998) to 34.3% (1999–2002) [1, 7]. Furthermore, data from the North American Pediatric Renal Trials and Collaborative Studies (NAPRTCS) indicate that peritonitis is the leading cause of modality switch among children undergoing CAPD [8].

The epidemiologic triad—comprising host, environment and agent—serves as a classic model for studying infectious diseases and their interactive dynamics that culminate in specific health outcomes. However, literature reveals that existing studies on peritonitis in CAPD patients have rarely examined these three components as an integrated system. Most research has focused on the host as a contributing factor, often assuming uniformity in infectious agents [7, 9–12]. Other studies emphasize the host’s psychosocial or environmental context [13–15]. To date, no research has concurrently investigated all three aspects of this infectious disease model, which is crucial for estimating risk while controlling for confounding variables [16]. In a country like Indonesia—particularly West Java—where socioeconomic disparities and dietary patterns vary widely due to urbanization, transmigration, and uneven regional development, examining these factors is also essential. Despite the known variability in household income, education levels, and food accessibility, there has been limited investigation into how these disparities intersect with CAPD outcomes. Furthermore, CAPD remains underutilized nationally, accounting for only 2% of renal replacement therapy modalities in ESRD patients as of 2021 [17]. This study aimed to determine the number of pediatric patients undergoing CAPD at our institution and to evaluate how host and environmental factors—including socioeconomic conditions and dietary diversity—contribute to CAPD-related peritonitis, while descriptively characterizing the causative microorganisms involved within an epidemiologic triad framework.

## Materials and methods

### Study design and setting

This study was designed as a retrospective cohort study, utilizing medical record data and direct patient contact to assess the clinical outcomes of pediatric patients with End-Stage Renal Disease (ESRD) undergoing Continuous Ambulatory Peritoneal Dialysis (CAPD). Ethical approval was obtained from the Ethics Committee of Hasan Sadikin General Hospital (approval number: DP.04.03/D.XIV.6.5/95/2024). The study population included all pediatric patients diagnosed with ESRD who initiated CAPD at Hasan Sadikin General Hospital between January 1, 2019, and December 31, 2024. Patients were identified through the hospital’s medical records system based on their CAPD initiation dates within the specified timeframe.

### Data collection

Data were collected from two primary sources: the patients’ electronic and/or paper medical records, and structured phone interviews with the patients or their legal guardians.

#### Medical record data

A standardized data extraction form was developed and used to collect the following information:

- Demographic Variables: Patient age, sex, and ethnicity.
- Clinical Variables: underlying cause of ESRD, complications during CAPD therapy such as peritonitis and other mechanical problems and date of CAPD insertion. Peritonitis was defined in accordance with the International Society for Peritoneal Dialysis (ISPD) guidelines, which require the presence of at least two of the following: (1) abdominal pain or cloudy peritoneal effluent; (2) elevated white blood cell count in dialysate; or (3) positive culture of dialysate fluid.
- Microbiological Data: For each episode of peritonitis, the type of causative microorganism was recorded based on peritoneal fluid culture.

#### Phone interview data

Structured telephone interviews were conducted with patients or their guardians to supplement and update clinical records with current information and household conditions. The following variables were collected:

- Patient Status: Current vital status (alive or deceased), date of death if applicable.
- Socioeconomic Variables: Father’s and mother’s education levels (categorized as primary, secondary, or higher education), paternal occupation (e.g., laborer, driver, civil servant, private employee, entrepreneur), parent’s working hour and monthly wage.
- Household Environmental and Nutritional Data: Household size (number of residents), Source of drinking water (e.g., well, municipal, or bottled), floor type (cement or ceramic), presence of livestock or pets in the household and dietary diversity. Dietary diversity was assessed using caregiver-reported variation in routinely consumed food groups, including carbohydrates, proteins, vegetables, fruits, and dairy products. Patients were categorized into low or moderate/high dietary diversity based on the number and variability of food groups consumed regularly.

Data collection, extraction, and interview processes were conducted by urology residents. A small number of discrepancies were encountered, primarily involving incomplete dates, differences between caregiver recall and medical records, or unclear socioeconomic categorization. These discrepancies were resolved through re-review of the original medical records and discussion between investigators. If disagreement persisted, a third reviewer independently evaluated the data and consensus was reached collectively to ensure consistency and data accuracy.

### Statistical analysis

Descriptive statistics were used to summarize baseline characteristics. Categorical variables were compared using the Chi-square or Fisher’s exact test, and continuous variables were analyzed using the Mann–Whitney U test as appropriate. Peritonitis rates were calculated per 100 person-months. For survival analysis, only the first episode of peritonitis was included in Kaplan–Meier estimation to evaluate peritonitis-free survival and minimize bias from recurrent events within individual patients. A p-value < 0.05 was considered statistically significant. All analyses were conducted using SPSS version 26.

## Results

Of the 46 pediatric patients undergoing CAPD identified for this study, 35 could be contacted and included in the final analysis. The most common age group was 12–17 years (51.4%), with a predominance of males (65.7%) and mostly Sundanese ethnicity (88.6%). The most common underlying renal disease category was glomerular disease (77.1%), with unclassified nephrotic syndrome being the most common subtype (40.0%). Although 11 patients could not be contacted for interview-based assessment, baseline medical record data showed a generally similar distribution of underlying renal diseases, predominantly glomerular disorders. Outcomes during the study period included peritonitis, CAPD technique-related complications, continuation of CAPD without major complications, and mortality. Survival analyses were based on time to first peritonitis episode. Peritonitis occurred in 11 patients (31.4%). CAPD was ineffective in 12 (34.3%), mainly due to catheter-related issues, while 12 (34.3%) continued CAPD during the observation period without major infectious or mechanical complications requiring intervention. Overall, 10 patients (28.6%) died, and 25 (71.4%) survived. At the last follow-up, 18 patients remained on CAPD and 7 patients transitioned to maintenance hemodialysis. None underwent kidney transplantation. Baseline demographic and clinical characteristics are summarized in Table 1.

**Table 1:** Association between baseline characteristics of pediatric patients undergoing CAPD and the occurrence of peritonitis

Characteristics	*N* = 35
Demography	
Age category, n (%)	
0–12 year	17 (48.6)
> 12–17 year	18 (51.4)
Sex, n (%)	
Male	23 (65.7)
Female	12 (34.3)
Ethnicity, n (%)	
Sundanese	31 (88.6)
Javanese	4 (11.4)
Underlying disease(s), n (%)	
Glomerular disease	
Unclassified nephrotic syndrome	14 (40.0)
Unclassified glomerulonephritis	2 (5.7)
Rapid Progressive Glomerulonephritis	3 (8.6)
Minimal Change Disease	1 (2.9)
Lupus nephritis	4 (11.4)
Juvenile idiopathic arthritis related glomerulonephritis	1 (2.9)
Focal segmental glomerulonephritis	1 (2.9)
CAKUT	
Reflux nephropathy	3 (8.6)
Chronic pyelonephritis	5 (14.3)
Unknown	1 (2.9)
Socioeconomic Status	
Main caregiver, n (%)	
Mother	29 (82.9)
Others	6 (17.1)
Father’s education, n (%)	
Elementary/junior high school	11 (31.4)
Senior high school/university	24 (68.6)
Mother’s education, n (%)	
Elementary/junior high school	17 (48.6)
Senior high school/university	18 (51.4)
Father’s occupation, n (%)	
Civil worker/employee/entrepreneur	21 (60.0)
Labourer/driver	14 (40.0)
Parents’ working hour, Median (Min – Max)	8 (5–24)
Monthly wage	
< Rp 4.000.000	15 (42.9)
≥Rp 4.000.000	20 (57.1)
Environment and Nutrition	
Handwash, n (%)	35 (100)
Diet diversity, n (%)	
Low	13 (37.1)
Moderate/High	22 (62.9)
Source of drinking water, n (%)	
Well water	19 (54.3)
Municipal/bottled water	16 (45.7)
Number of household member, Median (Min – Max)	4 (3–6)
Presence of livestock/pets, n (%)	
Yes	7 (20.0)
No	28 (80.0)
Floor type, n (%)	
Cement tiles	3 (8.6)
Ceramic tiles	32 (91.4)
Outcomes	
CAPD outcome, n (%)	
Peritonitis	11 (31.4)
Ineffective	12 (34.3)
No complications	12 (34.3)
Mortality, n (%)	
Yes	10 (28.6)
No	25 (71.4)

### Association between baseline characteristics and peritonitis

Among the 35 patients, 11 (31.4%) developed peritonitis. No significant associations were found between peritonitis and age (*p* = 0.803), sex (*p* = 1.000), or ethnicity (*p* = 0.575). While the distribution of underlying diseases did not differ significantly (*p* = 0.298), autoimmune glomerular diseases such as lupus nephritis and RPGN were more common in the peritonitis group. Staphylococcus aureus was the most frequently isolated pathogen (45.4%), followed by Staphylococcus epidermidis (27.2%), Pseudomonas aeruginosa (18.1%), and Escherichia coli (9.0%). Gram-positive bacteria were the dominant causative agents. Associations between socioeconomic and environmental variables and peritonitis are presented in Table 2, 3 and 4).

**Table 2:** Association between baseline characteristics and peritonitis incidence among pediatric CAPD patients. Statistical significance determined by Fisher’s exact or Mann–Whitney U tests

Characteristics	Peritonitis *n* = 11	Non-Peritonitis *n* = 24	*P* value
Demography			
Age category, n (%)			0.803^a^
0–12 year	5 (45.5)	12 (50)	
> 12–17 year	6 (54.5)	12 (50)	
Sex, n (%)			1.000^b^
Male	7 (63.6)	16 (66.7)	
Female	4 (36.4)	8 (33.3)	
Ethnicity, n (%)			0.575^b^
Sundanese	9 (81.8)	22 (91.7)	
Javanese	2 (18.2)	2 (8.3)	
Underlying disease(s), n (%)			0.298^a^
Glomerular disease			
Unclassified nephrotic syndrome	3 (27.3)	11 (45.8)	
Unclassified glomerulonephritis	0 (0)	2 (8.3)	
Rapid Progressive Glomerulonephritis	2 (18.2)	1 (4.2)	
Minimal Change Disease	0 (0)	1 (4.2)	
Lupus nephritis	3 (27.3)	1 (4.2)	
Juvenile idiopathic arthritis related glomerulonephritis	1 (9.1)	0 (0)	
Focal segmental glomerulonephritis	0 (0)	1 (4.2)	
CAKUT			
Reflux nephropathy	1 (9.1)	2 (8.3)	
Chronic pyelonephritis	1 (9.1)	4 (16.7)	
Unknown	0 (0)	1 (4.2)	
Socioeconomic Status			
Main caregiver, n (%)			0.352^b^
Mother	8 (72.7)	21 (87.5)	
Others	3 (27.3)	3 (12.5)	
Father’s education, n (%)			0.046^a^*
Elementary/junior high school	6 (54.5)	5 (20.8)	
Senior high school/university	5 (45.5)	19 (79.2)	
Mother’s education, n (%)			0.632^a^
Elementary/junior high school	6 (54.5)	11 (45.8)	
Senior high school/university	5 (45.5)	13 (54.2)	
Father’s occupation, n (%)			0.011^b^*
Civil worker/employee/entrepreneur	3 (27.3)	18 (75)	
Labourer/driver	8 (72.7)	6 (25)	
Parents’ working hour, Median (Min – Max)	8 (5–13)	8 (6–24)	0.899^c^
Monthly wage			0.144^b^
< Rp 4.000.000	7 (63.6)	8 (33.3)	
≥Rp 4.000.000	4 (36.4)	16 (66.7)	
Environment and Nutrition			
Diet diversity, n (%)			0.007^b^*
Low	8 (72.7)	5 (20.8)	
Moderate/High	3 (27.3)	19 (79.2)	
Source of drinking water, n (%)			0.027^b^*
Well water	9 (81.8)	10 (41.7)	
Municipal/bottled water	2 (18.2)	14 (58.3)	
Number of household member, Median (Min – Max)	5 (3–6)	4 (3–6)	0.388^c^
Presence of livestock/pets, n (%)			0.652^b^
Yes	3 (27.3)	4 (16.7)	
No	8 (72.7)	20 (83.3)	
Floor type, n (%)			0.227^b^
Cement tiles	2 (18.2)	1 (4.2)	
Ceramic tiles	9 (81.8)	23 (95.8)	
Outcome			
Mortality, n (%)			0.120^b^
Yes	1 (9.1)	9 (37.5)	
No	10 (90.9)	15 (62.5)	

**Table 3:** Causative agents in peritonitis patients

Causative Agents	Peritonitis *N* = 11
*Staphylococcus aureus*	5 (45.4)
*Staphylococcus epidermidis*	3 (27.2)
*Pseudomonas aeruginosa*	2 (18.1)
*E. coli*	1 (0.90)

**Table 4:** Number of events, peritonitis rate, and mean survival of peritonitis episodes based on characteristics in pediatric patients undergoing CAPD

Characteristics	Total Events	Peritonitis Rate per 100 person-Month (95% CI)	Mean Survival Month (95% CI)	*P* value
All subjects	11	4.5 (2.5–8.2)	17.9 (11.1–24.1)	
Socioeconomic Status				
Father’s education, n (%)				
Elementary/junior high school	6	7.2 (3.2–16.1)	12.4 (6.2–18.6)	0.292
Senior high school/university	5	3.1 (1.3–7.6)	24.5 (16.6–32.4)	
Father’s occupation, n (%)				
Civil worker/employee/entrepreneur	3	2.1 (0.7–6.5)	26.5 (17.6–35.3)	0.079
Laborer/driver	8	8.2 (4.1–16.3)	11.3 (5.7–16.8)	
Monthly wage				
< Rp 4.000.000	7	7.3 (3.4–15.3)	12.3 (6.4–18.2)	0.240
≥Rp 4.000.000	4	2.7 (1.0–7.3)	24.0 (11.1–24.7)	
Environment and Nutrition				
Diet diversity, n (%)				
Low	8	10.8 (5.4–21.6)	8.9 (3.8–14.0)	0.021*
Moderate/High	3	1.8 (0.6–5.5)	27.6 (20.1–35.2)	
Source of drinking water, n (%)				
Well water	9	8.2 (4.3–15.7)	10.8 (5.6–15.9)	0.069
Municipal/bottled water	2	1.5 (0.4–6.1)	28.9 (20.5–37.2)	
Floor type, n (%)				
Sement tiles	2	5.4 (1.4–21.6)	17 (3.3–30.7)	0.923
Ceramic tiles	9	4.4 (2.3–8.4)	19.6 (12.0–27.3)	

*p < 0.05 indicates statistical significance

### Socioeconomic factors

Father’s education was significantly associated with peritonitis (*p* = 0.046): 54.5% of affected children had fathers with only elementary or junior high education, compared to 20.8% among those with higher-educated fathers. Paternal occupation was also significant (*p* = 0.011), with peritonitis more frequent among children of laborers or drivers (72.7%) compared to those whose fathers held more stable jobs (27.3%). While not statistically significant, lower monthly income (< IDR 4,000,000) was more prevalent in peritonitis cases (63.6%, *p* = 0.144).

### Environmental and nutritional factors

Low dietary diversity was significantly associated with peritonitis (*p* = 0.007), affecting 72.7% of cases. Similarly, use of well water as the primary drinking source correlated with higher peritonitis rates (81.8%) compared to municipal/bottled water (18.2%) (*p* = 0.027). Other environmental factors, such as household size, livestock, and floor type, were not significantly associated.

### Mortality analysis

Although not statistically significant (*p* = 0.120), mortality was paradoxically higher in the non-peritonitis group (37.5%) than in the peritonitis group (9.1%).

### Peritonitis rate and survival

Survival analyses in this study specifically referred to peritonitis-free survival, defined as the time from CAPD initiation to the first documented peritonitis episode. The overall peritonitis rate was 4.5 per 100 person-months (95% CI: 2.5–8.2), with a mean peritonitis-free survival time of 17.9 months (95% CI: 11.1–24.1). Subgroup trends showed higher peritonitis rates and shorter survival among children with less-educated fathers (7.2 vs. 3.1; 12.4 vs. 24.5 months, *p* = 0.292) and those whose fathers were laborers/drivers (8.2 vs. 2.1; 11.3 vs. 26.5 months, *p* = 0.079). Likewise, lower family income showed a trend toward worse outcomes (*p* = 0.240).

### Environmental/nutritional impact on survival

Dietary diversity was the only variable significantly associated with both peritonitis rate and survival (*p* = 0.021). Children with low dietary diversity had a peritonitis rate of 10.8 per 100 person-months and mean survival of 8.9 months, compared to 1.8 and 27.6 months for those with moderate/high diversity. Well water use showed a near-significant association with higher peritonitis rates and lower survival (*p* = 0.069). Floor type had no effect on outcomes (*p* = 0.923).

### Kaplan–Meier analysis

**Fig. 1:**
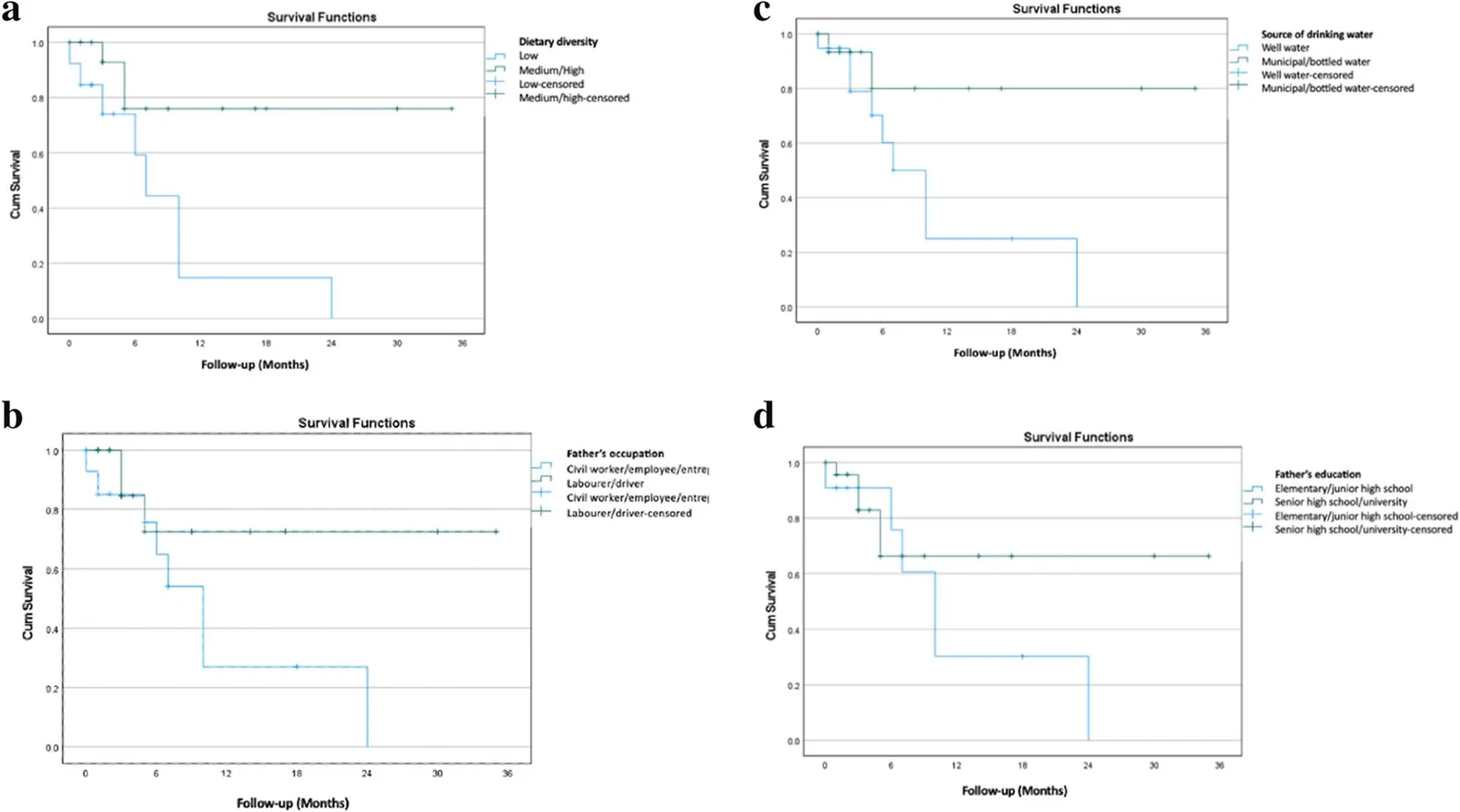
**a** Kaplan-Meier curve of peritonitis incidence based on dietary diversity in paediatric patients undergoing CAPD. **b** Kaplan-Meier curve of peritonitis incidence based on drinking water source in paediatric patients undergoing CAPD. **c** Kaplan-Meier curve of peritonitis incidence based on occupation in pediatric patients undergoing CAPD. **d** Kaplan-Meier curve of peritonitis incidence based on father's education level in pediatric patients undergoing CAPD.

Survival curves confirmed these patterns. Children with low dietary diversity showed steeper declines in peritonitis-free survival, reaching near-zero survival at 24 months (*p* = 0.021), while those with moderate/high diversity maintained high survival. Well water users showed rapid decline in peritonitis-free survival within 6–12 months, with only ~ 20% surviving at 24 months, compared to stable curves for municipal/bottled water users (*p* = 0.069). Children of laborers/drivers also showed early survival drop-off, while those with fathers in more stable occupations had prolonged survival (*p* = 0.079). Similarly, children with less-educated fathers had lower survival, consistent with earlier findings (*p* = 0.046), suggesting education may influence CAPD care quality and infection prevention (Fig. 1).

## Discussion

This study evaluated peritonitis risk through the lens of the epidemiologic triad, identifying key host, agent, and environmental factors associated with peritonitis in pediatric CAPD patients. The findings indicate that low dietary diversity (host factor), use of well water as the primary water source (environmental factor), and lower paternal education and occupation status (socioeconomic/environmental factors) are linked to higher peritonitis incidence and shorter peritonitis-free survival. These results are largely consistent with global and regional literature, while providing novel insights into how multiple domains interact to influence infection risk.

### Host factors

Nutritional status emerged as a critical host determinant. Children with low dietary diversity experienced a significantly higher peritonitis rate and markedly shorter peritonitis-free survival than those with more varied diets. This aligns with global evidence that malnutrition predisposes dialysis patients to infections [18]. Prior studies have noted that poor nutritional indicators (e.g. low serum albumin or protein intake) correlate with higher peritonitis risk [18, 19]. Our results extend these observations by using dietary diversity as a proxy for nutrition, underscoring that inadequate diet quality in pediatric CKD can impair immune defenses and wound healing, thereby increasing susceptibility to peritoneal infections. Furthermore, gut microbiota plays a role in peritoneal dialysis-related infections, and nutrition is a known influencer of this microbiota. In this study, low dietary diversity was significantly associated with peritonitis (*p* = 0.007), with 72.7% of cases occurring in patients with poor diet variety. This association may reflect the impact of gut dysbiosis, a common condition in end-stage kidney disease (ESKD) patients, where beneficial bacteria are reduced and pathogenic strains proliferate. A limited diet narrows the spectrum of microbial substrates, reducing microbiome diversity and resilience, and promoting the dominance of harmful organisms linked to systemic inflammation and infection. Gut dysbiosis may contribute to the development and progression of renal, metabolic, and inflammatory diseases. In peritoneal dialysis (PD) patients, increased uremic toxin-producing bacteria and reduced short-chain fatty acid-producing bacteria can impair intestinal barrier function, allowing microorganisms and toxins to enter the bloodstream and worsen peritoneal complications [20]. Nevertheless, dietary diversity should be interpreted cautiously as a surrogate marker rather than a direct measurement of nutritional adequacy. Detailed assessments of calorie intake, protein intake, anthropometric parameters, and biochemical nutritional markers were not consistently available in this retrospective cohort. Therefore, it remains possible that the observed association reflects broader protein-energy malnutrition, food insecurity, or socioeconomic disadvantage rather than an independent effect of dietary diversity itself. Future prospective studies incorporating comprehensive nutritional assessment are needed to better clarify the relationship between nutritional status and CAPD-related peritonitis. Future studies should also incorporate objective gastrointestinal variables such as constipation and bowel habits, which may more directly reflect bacterial translocation risk in PD-related peritonitis.

Notably, our cohort’s most common underlying CKD etiologies were glomerular diseases; although we did not find a statistically significant difference in peritonitis by disease type, the peritonitis group had more autoimmune conditions (lupus nephritis, RPGN), mirroring report that lupus nephritis patients have intrinsically higher infection risk [21]. Additionally, detailed longitudinal data regarding immunosuppressive therapy exposure were not consistently available, limiting further assessment of its contribution to infection susceptibility in patients with autoimmune renal disease. Meanwhile, results on age as risk factor were inconsistent, we observed no age or sex differences in peritonitis incidence. In two studies, there is an increased risk for peritonitis in older patients defined as > 65 or > 70 year, while in the other two it was found in patients under the age of 65 [22–25]. Overall, the host factors in our study highlight that a child’s biological and nutritional robustness significantly modulates peritonitis risk, emphasizing the need for diligent nutritional support and management of co-morbid conditions in pediatric PD programs.

### Agent factors

Although our analysis focused on risk factors rather than detailed microbiology, the causative organisms (the “agent” in the triad) provide context for how host and environmental factors translate into infection. Consistent with other PD data, Gram-positive bacteria (such as staphylococci and streptococci) are expected to account for a large proportion of peritonitis episodes [26]. Staphylococcus aureus was the most common causative agent (45.4%), followed by Staphylococcus epidermidis (27.2%), reflecting typical skin flora and highlighting the importance of proper hand hygiene and sterile technique during CAPD exchanges. In addition, the presence of environmental Gram-negative pathogens such as *Pseudomonas aeruginosa* (18.1%) and *Escherichia coli* (9.0%) were more closely associated with environmental contamination or enteric bacterial translocation.This finding aligns with the observed association between well water use and increased peritonitis risk, implying that poor water quality and inadequate household sanitation may contribute to the introduction of environmental pathogens during dialysis procedures. Nevertheless, due to the limited number of peritonitis episodes, organism-specific subgroup analysis could not be reliably performed. Indeed, studies in developing regions have noted higher rates of Gram-negative peritonitis compared to high-income countries, likely due to environmental contamination and hygiene challenges [27, 28]. It is plausible that these children’s peritonitis episodes included environmental organisms introduced during catheter care or handwashing.

### Environmental and socioeconomic factors

Perhaps the most striking associations were socio-environmental. Lower paternal education (only elementary/junior high) and labourer/driver occupations were significantly associated with peritonitis occurrence (both *p* < 0.05), and also corresponded to higher peritonitis rates and shorter survival on CAPD (though the survival differences did not reach *p* < 0.05). These findings resonate with other pediatric PD studies in developing regions. For example, a Chinese pediatric dialysis study reported that children with illiterate or non-parent caregivers and those with inadequate PD training had significantly higher peritonitis risk [29]. Similarly, a comprehensive risk factor review identified low socioeconomic status and psychosocial factors as valid predictors of PD-related peritonitis [30, 31]. In this study, we found that father’s education significantly influenced the incidence of peritonitis in children, likely due to its strong correlation with socioeconomic status, health literacy, and decision-making capacity within the household. Although mothers were the primary caregivers performing CAPD exchanges in most households, maternal education was not significantly associated with peritonitis in this cohort. In our sociocultural setting, fathers often play a central role in household financial support, healthcare access, and medical decision-making, which may indirectly influence adherence to CAPD care and infection prevention practices. Therefore, paternal education and occupation may function as broader indicators of household socioeconomic stability and health literacy. Fathers with only elementary or junior high school education may have limited access to stable, well-paying jobs, leading to reduced financial resources for timely medical care. Moreover, caregiver education likely affects the understanding and execution of sterile technique in home-based dialysis. A father or family member with limited education may have difficulty comprehending medical instructions or accessing and interpreting educational materials, which can lead to improper dialysis technique and breaches in aseptic procedure. In contrast, fathers with higher education levels are more likely to secure stable employment, possess better knowledge of health-related issues, and support adherence to proper dialysis protocols. Our data also suggest that children from socioeconomically disadvantaged families are more vulnerable to peritonitis, which is consistent with the broader literature linking poverty to higher infection rates in chronic dialysis [18, 30]. Importantly, we noted that monthly household income < IDR 4 million was more frequent in the peritonitis group (64% vs. ~ 40%) even though this difference was not statistically significant. The direction of this trend aligns with the education and occupation findings, reinforcing a socioeconomic gradient in peritonitis risk. Taken together, these environmental findings emphasize that the context in which a child undergoes CAPD – including the cleanliness of their living environment, water supply, and family resources – is pivotal in determining outcomes. Interventions aimed at caregiver training and support could therefore have a significant impact on reducing infections in this population. However, we acknowledge that the environmental variables evaluated in this study—such as parental occupation and drinking water source—represent indirect surrogate markers rather than direct measures of dialysis-related infection exposure. More specific environmental factors including hand hygiene practices, caregiver training quality, exchange room conditions, and aseptic technique adherence were not consistently available in this retrospective cohort and should be evaluated in future prospective studies.

### Interplay of triad factors

Our results support the concept that peritoneal dialysis-related peritonitis arises from a confluence of host susceptibility, agent pathogenicity, and environmental exposure. For example, a malnourished child (host factor) living in a household with poor water sanitation (environment) is at particularly high risk of an infection if exposed to bacteria (agent). Our study demonstrates that significant insights are gained by examining all three corners of this triad. We found that each domain contributed to the overall risk profile: host nutritional deficits made infections more likely to take hold, environmental lapses increased the chance of microbial contamination, and the organisms involved can exploit these vulnerabilities. Notably, the fact that low dietary diversity was the only factor independently associated with both higher peritonitis incidence and shorter peritonitis-free survival suggests it may act as a permissive factor – i.e. an impaired host immune defense allows any contamination (agent/environment) to more readily progress to clinical peritonitis and may play a role in gut dysbiosis. Meanwhile, the near-significant survival impacts of water source and parental occupation indicate that these environmental factors accelerate time to infection, likely by increasing exposure load or reducing adherence to preventive measures.

Given the significant impact of parental education—particularly the father’s—on peritonitis risk, comprehensive education for both parents during clinic visits is essential. Health education should not be limited to mothers or primary caregivers, as fathers often influence household decision-making and resource allocation. Therefore, both parents must actively learn proper peritoneal dialysis techniques, infection prevention, and early complication signs. Furthermore, regular multidisciplinary follow-up after initiating CAPD—both with pediatric and urology clinics—is crucial to ensure correct home dialysis practice and timely correction of any technique, hygiene, or equipment issues, ultimately reducing infection-related complications like peritonitis. These dynamics underscore that prevention strategies must be multifactorial; focusing on a single aspect (such as just antibiotic prophylaxis against certain bacteria) without improving nutrition and home hygiene would leave substantial residual risk.

This study found a paradox in which mortality was higher in the non-peritonitis group (37.5%) compared to the peritonitis group (9.1%), although the difference was not statistically significant (*p* = 0.120). This unexpected pattern may be explained by several factors. First, the small sample size and exclusion of 11 unreachable patients may have introduced selection bias and reduced statistical power, leading to wide confidence intervals and limiting the detection of significant differences. Second, peritonitis often presents with clear symptoms such as abdominal pain, fever, or cloudy dialysate, which may prompt faster medical recognition and intervention. This heightened awareness among parents or caregivers can lead to more timely treatment and closer monitoring, potentially improving outcomes. In contrast, the non-peritonitis group in this study included patients who experienced serious complications such as mechanical failure, catheter obstruction, or intra-abdominal adhesions—conditions that may not present with overt infectious symptoms but can result in severe clinical deterioration and contribute to early mortality. Additionally, these complications may have necessitated the discontinuation of peritoneal dialysis, leading to treatment gaps or suboptimal transitions to alternative modalities. Survival bias may also play a role, as patients who developed peritonitis may have remained on dialysis longer, allowing time for the complication to occur, while others in the non-peritonitis group may have died earlier. These paradoxical findings likely reflect both the clinical complexity of non-infectious complications and methodological limitations. This single-center, retrospective study with a small sample size (35 patients) limits generalizability and introduces potential for bias. The absence of an a priori sample size calculation and the relatively small sample size may have reduced statistical power, increasing the possibility of type II error, particularly in subgroup and survival analyses. Due to the limited sample size and number of peritonitis events, only bivariate analyses were performed. More advanced multivariable or mixed-model methodologies were not conducted because they would likely produce unstable estimates and substantial risk of model overfitting. Therefore, the present analyses should be interpreted as exploratory. In addition, recurrent peritonitis episodes and dialysis technique survival were not separately analyzed because of the limited sample size and small number of recurrent events. Reliance on medical records meant that key factors such as PD technique, hand hygiene, and actual dietary intake were approximated using proxy variables, which may not fully capture clinical realities. Additionally, data on dietary diversity and water source may not accurately reflect true nutritional status or sanitation practices. Moreover, although this study adopted an epidemiologic triad framework, the agent component was evaluated descriptively through microbiological profiles and not through detailed analysis correlating specific organisms with baseline host or environmental variables. Therefore, the interaction between all three triad components could not be fully established. Culture-negative cases and the absence of microbial typing further restricted in-depth analysis of causative agents.

Despite these limitations, our findings are broadly comparable to those reported in other parts of Southeast Asia, with some differences in magnitude. The overall peritonitis rate in our center was 4.5 episodes per 100 patient-months (~ 0.54 episodes/patient-year), which is somewhat higher than the rates of 0.303/patient-year reported in large registries from global data [32]. It is also higher than the 0.42 episodes/patient-year recently reported in Jakarta’s pediatric dialysis program [26], and substantially exceeds the 0.18 episodes/patient-year described in a Malaysian series [17]. These observations underscore the need for cautious interpretation and highlight the importance of larger, prospective studies to validate these findings and better understand both infectious and non-infectious outcomes in pediatric peritoneal dialysis.

## Conclusion

This study highlights that peritonitis in pediatric CAPD patients arises from a complex interplay of nutritional, environmental, and socioeconomic factors. Low dietary diversity, poor water quality, and lower paternal education and occupation were associated with higher peritonitis risk and shorter peritonitis-free survival. Effective prevention in resource-limited settings therefore requires a multifaceted approach, including nutritional support, hygiene improvement, and comprehensive caregiver education, to improve outcomes and sustain CAPD as a viable bridging therapy for children with ESRD. Given the limited sample size and retrospective single-center design, these findings should be interpreted cautiously and validated in larger multicenter prospective studies.

## Supplementary Information

Supplementary Material 1.

## Data Availability

The datasets used and/or analysed during the current study are available from the corresponding author on reasonable request.

## Abbreviations

CAPD: Continuous Ambulatory Peritoneal Dialysis
CKD: Chronic Kidney Disease
ESRD: End-Stage Renal Disease
ISPD: International Society for Peritoneal Dialysis
PD: Peritoneal Dialysis
