# Supplementary material for: Evaluation of the epidemiologic triad in the incidence of peritonitis among pediatric patients with chronic kidney disease undergoing peritoneal dialysis: a single-center study in Indonesia
Source: BMC Pediatr. 2026 Jun 26;26:863. doi: 10.1186/s12887-026-07185-8 (PMC13573438; doi:10.1186/s12887-026-07185-8)
Supplement: Supplementary file 1 [file 12887_2026_7185_MOESM1_ESM.docx]

**Title**

**Evaluation of the Epidemiologic Triad in the Incidence of Peritonitis Among Pediatric Patients with Chronic Kidney Disease Undergoing Peritoneal Dialysis: A Single-Center Study**

**Authors and Affiliations**

Clarissa Tania^1^
Aaron Tigor Sihombing^1^

^1^Department of Urology, Hasan Sadikin Hospital, Universitas Padjadjaran, West Java, Indonesia

**Corresponding Author**

**Clarissa Tania** (corresponding author)
Department of Urology, Hasan Sadikin Hospital, Universitas Padjadjaran
Jl. Pasteur No.38, Bandung, West Java, Indonesia
Email: **clarissa23005@mail.unpad.ac.id**
Phone: **+6285281883608**

**Funding**

None

**Acknowledgements**

None

**Conflict of Interest**

The authors declare no conflict of interest.

**Ethics Approval**

The study was approved by the Ethics Committee of Hasan Sadikin General Hospital, Universitas Padjadjaran (approval number: [DP.04.03/D.XIV.6.5/95/2024]/

**Consent to Participate**

Written informed consent was obtained from all individual participants and/or their legal guardians.

**Consent to Publish**

Consent for publication was obtained from the participants and/or their guardians.

**Clinical Trial Number**

Clinical trial number: not applicable.

**Human Ethics and Consent to Participate Declaration**

All procedures involving human participants were performed in accordance with the ethical standards of the institutional and/or national research committee. Human Ethics and Consent to Participate declarations: included and applicable.

**Author Contributions**

Clarissa Tania: Conceptualization, methodology, data curation, writing – original draft preparation.
Aaron Tigor Sihombing: Supervision, validation, writing – review & editing.
All authors have read and approved the final version of the manuscript.
